# The HER2-Encoded miR-4728-3p Regulates ESR1 through a Non-Canonical Internal Seed Interaction

**DOI:** 10.1371/journal.pone.0097200

**Published:** 2014-05-14

**Authors:** Inga Newie, Rolf Søkilde, Helena Persson, Dorthe Grabau, Natalia Rego, Anders Kvist, Kristoffer von Stedingk, Håkan Axelson, Åke Borg, Johan Vallon-Christersson, Carlos Rovira

**Affiliations:** 1 BioCare, Strategic Cancer Research Program, Lund, Sweden; 2 CREATE Health, Strategic Centre for Translational Cancer Research, Lund, Sweden; 3 Department of Pathology, Skåne University Hospital, Lund, Sweden; 4 Department of Laboratory Medicine, Lund University Cancer Center, Lund, Sweden; 5 Department of Oncology and Pathology, Lund University Cancer Center, Lund, Sweden; CNRS, UMR7275, France

## Abstract

Since the early 1980s remarkable progress has been made in understanding the role of the HER2 locus in carcinogenesis, but many details of its regulatory network are still elusive. We recently reported the finding of 367 new human microRNA (miRNA) genes of which one, mir-4728, is encoded in an intron of the HER2 gene. Here, we confirm that the *HER2* oncogene is a bi-functional locus encoding the membrane receptor and a functional miRNA gene. We further show that miR-4728-3p has alternative functionalities depending on the region used for interaction with its target; the canonical seed between nucleotides 2–8 or a novel, more internal seed shifted to nucleotides 6–12. Analysis of public data shows that this internal seed region, although rare compared to the far more abundant canonical 2–8 seed interaction, can also direct targeted down-regulation by other miRNAs. Through the internal seed, miR-4728-3p regulates expression of estrogen receptor alpha, an interaction that would have remained undetected if classic rules for miRNA-target interaction had been applied. In summary, we present here an alternative mode of miRNA regulation and demonstrate this dual function of the HER2 locus, linking the two major biomarkers in breast cancer.

## Introduction

Human epidermal growth factor receptor 2 (Erbb2/HER2, hereafter called HER2) and estrogen receptor alpha (ESR1) are the most important prognostic and treatment predictive biomarkers in breast cancer (BC) and they are the most widely used therapeutic targets for this disease [1], [2].The *HER2* oncogene is amplified in 15–20% of all invasive BCs, leading to overexpression of the gene. Its tyrosine kinase activity triggers a signal transduction cascade that controls cell growth, proliferation and differentiation and is also associated with carcinogenesis in a range of epithelial cancers such as endometrial, lung, gastric, ovarian, esophageal, and bladder cancers as well as medulloblastoma and glioma (For a review see Zaczek *et al.* [*3*], Moasser *et al.* [*4*]; Olayioye *et al.* [*5*]). Due to its usefulness as a prognostic biomarker and for targeted therapy, *HER2* amplification in BC is routinely tested for in clinical laboratories (tumors being classified as amplified ‘HER2+’ or non-amplified ‘HER2-’). More than 70% of all BCs overexpress ESR1 as judged by immunohistochemistry (ER+ tumors) and expression of ESR1 is highly predictive of clinical benefit from endocrine therapies such as treatment with estrogen receptor modulators or aromatase inhibitors. Of note, HER2 amplification is associated with poor response to endocrine therapy.

*HER2* amplification-driven carcinogenesis implies protein overexpression and increased signal transduction, but the basal requirement for transformation is transcriptional overexpression [4]. This may suggest that the oncogenic activity is not solely associated with mitogenic signaling [6]. Indeed, we recently identified mir-4728 [7], a microRNA (miRNA) encoded in intron 24 of the *HER2* gene. Simultaneous production of *HER2* mRNA and the miRNA implies that this locus may have functions that are independent of signal transduction through the HER2 receptor.

Bioinformatic target gene prediction is a frequently used method for assessing the potential functions of miRNAs. Comparative sequence analysis and experimental studies have shown that a perfect match between the target site and nucleotides (nt) 2–8 from the 5' end of the miRNA, the seed region, determines miRNA target specificity [8]. Most algorithms for target gene prediction are based on this type of interaction [9], although perfect seed paring is not always the main determinant for repression. For instance, the prototypical miRNAs of *C. elegans,* lin-4 and let-7, also function in target gene regulation with imperfect seed pairing [10], [11]. Base-pairing beyond the seed region can in fact be crucial for target interaction [12], not only by compensating for imperfect seed pairing [13], [14], but also by making a larger contribution than the seed to duplex stability, as exemplified by a miR-122 site in the human hepatitis C virus [15]. In fact, global analysis of Argonaute protein (AGO) interactions across the transcriptome has recently uncovered evidence of exceptions to the seed rule [16]–[18]. In mouse brain, non-canonical miRNA base-pairing represents ∼15% of all AGO-associated interactions [19] and as much as 43% of all miR-155 target sites do not follow seed rules in T cells [16].

With this in mind, we decided to study miR-4728-3p function without applying prior knowledge of the interaction mode or requirements for evolutionary conservation. By investigating the effects of miR-4728-3p on global expression data we found that it functions as a bimodal miRNA, controlling different target gene sets depending on the region used for interaction; involving either a canonical seed in positions 2–8 or nt 6–12 of the miRNA. Since this region shares functional characteristics with the canonical seed, we called it an internal seed. We furthermore demonstrate that miR-4728-3p down regulates expression of ESR1 through an internal seed interaction. This clinically very relevant interaction would have passed undetected if current rules for miRNA function had been applied for miR-4728-3p target prediction. In summary, our results add a new layer of functional complexity to the *HER2* oncogene, expand the repertoire of regulatory miRNA-target gene interactions and demonstrate the existence of a direct RNA-RNA crosstalk between the two major therapeutic breast cancer biomarkers.

## Materials and Methods

### Cell culture and Transfections

All cell lines were purchased from ATCC and used at low passage numbers. Cells were cultured as reported previously [20] except that for MCF7, insulin was added at 10 µg/ml.

Transfections were performed with Lipofectamine 2000 (Life Technologies) following the manufacturer's instructions. All oligonucleotides and miRNA mimics were transfected at 25 nM or 100 nM unless stated otherwise. Antisense oligonucleotides contained 2′ O-methyl modifications and were from IDT DNA Technologies. Non-targeting siRNA control was from Thermo Scientific. All miRNA mimics were purchased from Qiagen; the customized mimics corresponding to the mature sequence of miR-4728-3p (25 nt) and a 25 nt control.

For luciferase reporter assays, part of the 3′ UTR of the target gene was cloned into pmirGlo Dual-Luciferase miRNA Target Expression Vector (Promega) and transfected at 400 ng in 12-well plates.

### Northern blot analysis

Total RNA from miR-4728-3p transfected and untransfected MCF10A cells was prepared with TriZol reagent (Life Technologies) according to the manufacturer's instructions. Total RNA (∼5 µg) was separated on a denaturing 15% PAGE-urea gel and transferred onto GeneScreen Plus membrane (Perkin Elmer). Blots were hybridized with ^32^P-labeled miR-4728-3p probe for 16 hours in 7% SDS, 200 mM Na_2_HPO_4_ (pH 7.2), 1 mM EDTA and 1x Denhardt's solution at 40°C. After four washes in 3×SSC, 25 mM NaH_2_PO_4_ (pH 7.5), 5% SDS and 5X Denhardt's solution for 10 min at room temperature we performed a stringent wash in 1xSSC: 1% SDS for 5 min at 40°C. Then, the membrane was exposed for 6 h and analyzed in a Fuji phosphor imager.

### Western Blot

Cells were synchronized by serum starvation (1% FBS) for 6 h prior to harvest and stimulated for 5 min with EGF. Unless stated otherwise, cells were harvested 30 h post transfection on ice in RIPA buffer (10 mM Tris-HCl pH 7.4, 150 mM NaCl, 1 mM EDTA, 0.1% SDS, 1% Triton X-100, and 1% sodium deoxycholate) supplemented with Complete Protease Inhibitor Mixture tablets (Roche Diagnostics). Lysates were clarified by centrifugation and protein concentrations were determined by BCA Protein Assay kit (Thermo Scientific). Equal amounts of crude lysates were separated by SDS-PAGE on 4–12% bis-tris gels and proteins were transferred to a PVDF membrane (both Life Technologies) for immunoblot analysis. Membranes were then blocked and probed with the following primary antibodies: ESR1 (Santa Cruz sc-543), ERBB2 (Sigma-Aldrich HPA001383), pMAPK, pAKT, MAPK, AKT (Cell Signaling: 4370, 4060, 9102, 2938) and tubulin (abcam ab7291) according to the manufacturers' instructions. HRP-conjugated secondary antibodies (abcam) were visualized with ECL (GE Healthcare or Santa Cruz) and staining intensity was determined using a FluorChem FC2 with AlphaView software (Cell Biosciences). For quantification, membranes were stained with Coomassie R-350 (GE Healthcare) and analyzed with ImageJ software as reported by Welinder and Ekblad [21]. Between hybridizations, membranes were stripped with Restore Plus Western Blot Stripping Buffer (Thermo Scientific) for 10 min at room temperature and washed extensively in TBST before subsequent antibody incubations.

### Luciferase Reporter assays

For Luciferase Reporter assays, cells were transfected close to confluence, harvested 30 h post transfection and analyzed using the Dual-Luciferase Reporter Assay System (Promega) according to the manufacturer's instructions. Firefly luciferase activity was normalized to control Renilla luciferase and assays were performed at least in 5 biological replicates and technical triplicates. Significance analysis was performed with a 2-sided Student's t-Test.

### Microarray expression analysis

RNA was extracted with TRIZOL (Life Technologies) according to the manufacturer's instructions. RNA quantity and quality were assessed with NanoDrop ND 1000 spectrophotometer (NanoDrop Tech) and LabChip GX (Perkin Elmer) respectively before loading the samples on a HumanHT-12 v4.0 (mir-1 overexpression, non-targeting control) or HumanHT-12 v3.0 (all residual samples) Expression BeadChip (Illumina) in 4–6 biological replicates. Raw data is available at the GEO repository under accession number GSE55822.

All data were imported and normalized using the Base server (http://base.thep.lu.se). Empirical cumulative distribution function (ECDF) plots as introduced by Grimson *et al.* [*12*] were created with the array results. Downloaded 3′ UTR sequences from the SylArray [22] analysis were searched for words containing either an internal seed (IS) or canonical seed (CS). A single factor was generated for each array probe calling if it had 1 or more IS, 1 or more CS or 1 or more of both seed types. The log fold changes are plotted as in Grimson *et al.* to illustrate and compare the strength of the different seed types.

The RNAduplex 2.1.1 function [23] from the Vienna RNA Package 2.0 was used to calculate the energy of duplex structures formed between the microRNA mimic and the section of mRNA interaction. Values are given in (kcal/mol).

### qPCR and qRT-PCR

DNA and RNA were extracted using the AllPrep DNA/RNA FFPE Kit (Qiagen) on Formalin Fixed Paraffin Embedded (FFPE) tissue of 38 breast cancers, of which 19 were HER2+ and 19 HER2- tumors.

All samples were quantified by NanoDrop ND 1000 spectrophotometer (NanoDrop Tech). Poly(A) tailing and reverse transcription (RT) were conducted as follows: 100 ng of total RNA was used for cDNA synthesis with 1 µM universal RT primer, 1 unit (U) poly(A) polymerase, and 100 U MuLV reverse transcriptase (both New England Biolabs) in a 10 µl reaction of 1x poly(A) polymerase buffer.

For FFPE samples a mix of 1 µM reverse primers for mRNA targets were added to increase sensitivity. cDNA was diluted 1∶10 prior to qPCR reactions. For quantification, SYBR green reagents (Solis Biodyne or BioRad) were applied according to the manufacturers' instructions in 25 µl reactions on a CFX96 instrument (BioRad). Cycling conditions were: 15 min 95°C enzyme activation, followed by 45 cycles of 15 sec at 95°C, 30 sec at 60°C; with subsequent melting curve analysis. qRT-PCR expression data were normalized to selected reference genes (ACTB, RN7SL, RNU6, SNORD48, hsa-let-7a and hsa-miR-191) in qbasePLUS. DNA qPCR expression data were normalized to two control regions on chromosomes 3 and 6. HER2 RNA expression was measured with exon spanning primers comprising exon junctions 2–3 and 22–23, while ESR1 expression is given as average of two exon spanning primer pairs (to exon junctions 1–2 and 7–8) as well as a primer pair in the 3′ UTR. *HER2* DNA amplification was measured as amplification of exons 24 and 27. qPCR/qRT-PCR expression data were exported after normalization and plotted using R. Spearman correlation coefficients and p-values were calculated in R on all complete cases.

All primer sequences can be found in the Table S2.

### Ago CLIP

For Ago HITS CLIP cells were UV irradiated in a Stratalinker (Stratagene) twice with 400 mJ/cm^2^. Antibodies used for Ago IP were monoclonal Ago2 4G8 (Wako Chemicals) or anti-pan Ago clone 2A8 (Merck Millipore).

### Next Generation Sequencing

Sequencing libraries from purified RNA were prepared with NEBNext Multiplex Small RNA Library Prep Set for Illumina (New England Biolabs) according to the manufacturer's instructions and sequenced on Illumina HiSeq sequencer in paired end mode with 2×101 cycles.

Novoalign version 3 was used to align paired end reads from all sequencing libraries. Reads were first mapped against rRNA and pre-rRNA sequences to filter out contamination by rRNA fragments. Secondly the forward reads of unaligned sequences were processed to fasta files and collapsed to unique reads using the fastx_toolkit (http://hannonlab.cshl.edu/fastx_toolkit/). Unique reads were aligned to the human genome (hg19) with decoy sequences. Only reads with unique mappings were allowed in downstream analyses.

Reads mapping to miR-4728-3p were analyzed in the processing from the 5′ end. Both forward and reverse reads were analyzed and normalized to the total number of reads mapped to the human genome.

The Bioconductor [24] R package “chipseq” was used to compare the results of the different sequencing libraries.

## Results

### MicroRNA-4728-3p uses two different seed interactions

In our previous work we showed that the dominant mature form of mir-4728 is the one derived from the 3' arm of the precursor; miR-4728-3p [7]. To study its function, we transfected miR-4728-3p mimics into MCF 10A, a normal-like breast epithelial cell line normally not expressing mir-4728, and analyzed global effects on gene expression using microarrays. We confirmed the transfection efficiency by quantitative RT-PCR analysis on RNA co-immunoprecipitated with AGO2 (data not shown). Additionally, we transfected MCF 10A in a control experiment with a mimic of human miR-1, a well-characterized gene involved in the differentiation of smooth and skeletal muscles, normally not expressed in breast cell lines. 82% of the validated targets for miR-1 [25] expressed in MCF 10A were down regulated, confirming the accuracy of the experimental strategy.

**Figure 1:**
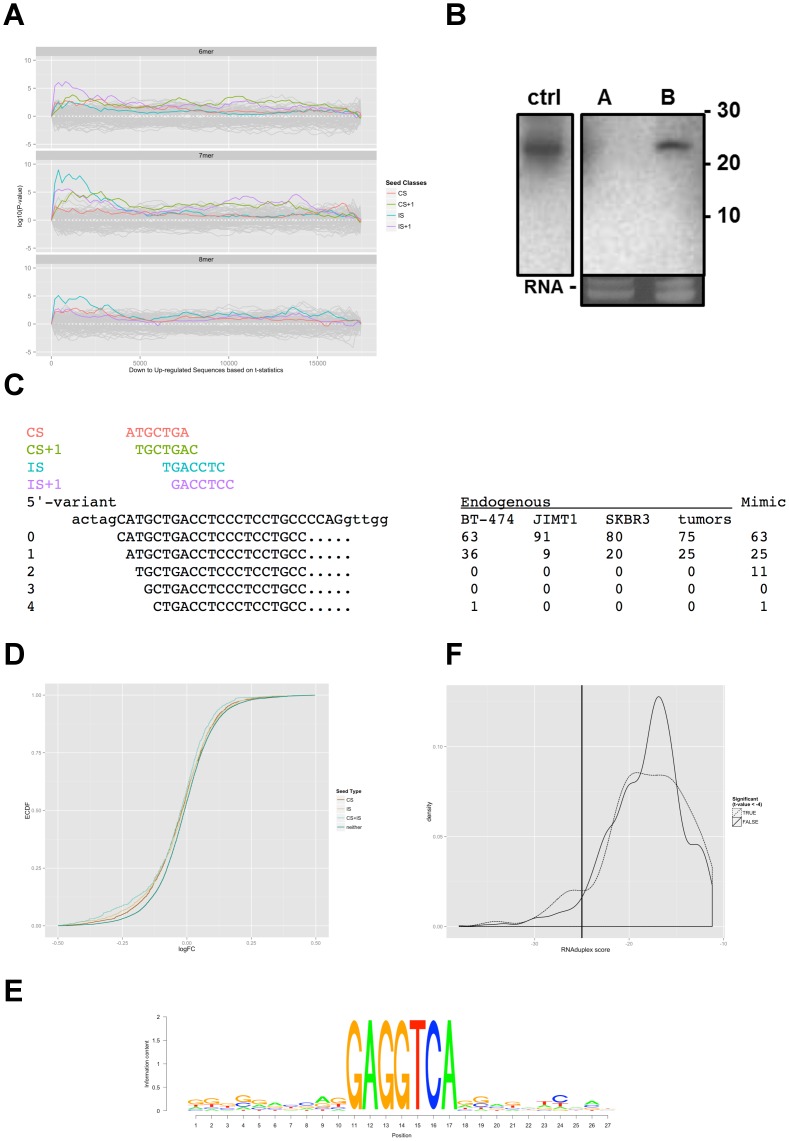
The main isoform of miR-4728-3p is 24-26nt long and interacts with its targets through an internal seed. **A.** SylArray enrichment landscape plots for 6-, 7- and 8-mer words (from top to bottom) for ranked genes from a microarray experiment of miR-4728-3p overexpression in MCF 10A. 3′ UTRs are sorted on the x-axis from most down (left) to up regulated (right). Significance of the words is given as log-transformed P-values, where over- and underrepresentation are shown on the positive and negative y axis, respectively. Highlighted are targets corresponding to canonical seed (CS, red), CS shifted by one base towards the miRNA'3′ end (CS+1, green), internal seed (IS, blue) and IS +1 (purple). CS+1 is below significance cut-off and it is not highlighted for 8-mer. **B.** Northern blot of total RNA from cells A) untransfected and B) transfected with miR-4728-3p mimics with a radiolabeled miR-4728-3p probe. Control lane shows signal from synthetic miR-4728-3p RNA and the ethidium bromide staining in the lower panel shows tRNA bands as loading control. **C.** Alignment of small RNA sequencing reads to miR-4728-3p genomic context. Positions of CS, CS+1, IS, and IS+1 are highlighted in color as in panel A above the alignment. Sequencing reads of mir-4728 in cell lines with endogenous expression (BT-474, JIMT1 and SKBR3), mimic-transfected MCF 10A (Mimic) and tumors are given to the right as percentages of total miR-4728-3p reads. **D.** Empirical cumulative distribution functions (ECDF) show effectiveness of CS and IS target sites. mRNA abundance after miRNA transfection in MCF 10A was monitored with microarrays. Distributions of changes for 3′ UTRs of mRNAs containing CS, IS, both, or neither are colored as denoted on the right. Each class contains the seed and the respective shifted seed (+1). **E.** Conservation plot. Top 250 down regulated genes from a microarray experiment of miR-4728-3p overexpression were filtered for IS and CS target sites respectively. Target sequence context of 10 bases on either side of the seeds was extracted and analyzed with multiple sequence alignment. The IS conservation plot shows conservation of the target site but not of surrounding nucleotides. **F.** Distribution of duplex energies of 3' UTRs containing IS target sites comparing genes down regulated by miR-4728-3p overexpression (t-statistics of <-4, TRUE) with unaffected genes (t-statistics ≥4, FALSE). Thermodynamic stability of hybrid formation between targets and miR-4728-3p was calculated using RNAduplex from the Vienna package. RNA duplex score is shown on the x-axis while target density is expressed on the y-axis. Folding stability is higher in a small number of regulated targets but otherwise similar to non-regulated targets, and only one motif with high duplex stability was part of a longer motif (10nt).

In lieu of applying routine target prediction algorithms, we then performed a motif search on microarray data from six biological replicates 32 hours after transfection of miR-4728-3p and controls including miR-1. We identified over-represented stretches of consecutive bases (“words”) in the 3' untranslated regions (UTRs) of genes from ranked gene lists and calculated the statistical significance of their enrichment using SylArray [22]. Unsurprisingly, the strongest signals detected among down regulated genes corresponded to words complementary to the transfected miRNAs. But while down regulation of genes carrying canonical seed (CS) 2–8 target sites was the most prominent feature in the miR-1 control experiment (Figure S1 in File S1, middle panel), CS matches were only the third most enriched word for miR-4728-3p. Here, the strongest signal upon overexpression was the word GAGGTCA, which instead matches the more internal region between residues 6–12 from the 5' end of the mature miRNA (Fig. 1A, middle panel). This sequence has no complementarity to any CS deposited in miRBase 20, suggesting the use of an alternative seed for miR-4728-3p. Due to its similar length and location with respect to the CS we named this putative new seed type internal seed (IS).

We speculated that a truncated miR-4728-3p, shortened by four nt at its 5' end, would convert positions 6–12 into a canonical nt 2–8 seed. Quality control of the synthetic mimics by mass spectrometry showed that more than 90% were full-length (personal correspondence with manufacturer) but their integrity could be affected after transfection. To exclude this possibility we performed a northern blot analysis with total RNA extracted from miR-4728-3p mimic transfected cells (Fig. 1B). This analysis showed that all mimics detected after transfection were in the 25 nt range as expected. To further clarify this point, we analyzed the distribution of sequenced miR-4728-3p 5′-ends obtained by crosslinking immunoprecipitation from AGO2 complexes (AGO-CLIP) in MCF 10A cells transfected with miR-4728-3p mimics. The two main 5′ isomiRs detected for miR-4728-3p were between 24–25 nt long and displayed the canonical 5′ end or a second one shortened by one base (Fig. 1C). CS and IS matches for the one nt shorter 5′ isomiRs were also detected as enriched by SylArray (labeled CS+1 and IS+1 in Figs. 1A and C) suggesting that the position of the IS, as is the case for CS, is determined by the distance to the 5′ end. Seed sites for the third most abundant 5′ variant (two nt shorter) were not significantly regulated in the array expression data and the presence of yet shorter 5′ isomiRs was negligible even if their presence cannot be completely excluded. We therefore concluded that the IS enrichment detected by SylArray was not caused by CS matches of a truncated miR-4728-3p mimic.

We then evaluated the possibility of the IS as a consequence of mere extension of CS base pairing. We found that the majority of down regulated 3′ UTRs with IS matches are devoid of CS sites. In fact, a cumulative distribution function of signals from array probes detecting 3′ UTRs carrying only CS matches (n = 1752), only IS matches (n = 1078) or both (n = 307) shows that the two seeds act independently of one another and that IS sites are sufficient for miR-4728-3p-guided repression (Fig. 1D). Interestingly, in this experiment targets with only IS sites are more down regulated than those with only CS matches in agreement with the SylArray enrichment results. The presence of an IS match also increases the level of repression for targets with CS matches causing an additive effect (Fig. 1D). These results support the existence of a functional internal seed region at positions 6–12 and suggest that miR-4728-3p is a bimodal miRNA that may alternate between seed regions 2–8 and 6–12 to specifically down regulate different target gene sets.

Next, we tested whether the GAGGTCA motif is part of longer regulatory motifs such as the centered site [26] which lacks seed pairing but has ∼11 consecutive base pairs that could overlap the IS. We analyzed word sizes from 5 to 9 nt among regulated targets, but only found significant enrichment for words between 6 nt (positions 6 to 11) and 8 nt (positions 6–13) centered on the IS match; all sharing the same 5′ position (Fig. 1A). Words longer than 8 nt were not overrepresented among down regulated genes (data not shown). This observation is analogous to the CS site of miR-1 in the control experiment (Fig. S1 in File S1). We also examined the nature of miR-4728-3p target gene interactions among the down regulated genes detected by microarrays. Among the top 250 down regulated genes (adjusted p-value < 0.01), 88 had IS 7-mer matches (GAGGTCA) in their 3′ UTRs. A similar analysis detected 169 3′UTRs with matches to the GAGGTC 6-mer. We extracted the 7-mer target sites together with their natural sequence context comprising 10 nt on either side of the target site. Figure 1E shows a graphical representation of the multiple sequence alignment of these fragments, confirming that the IS match is the only common motif.

To exclude the need of extensive compensatory base-paring outside the IS, we predicted the thermodynamic stability of hybrid formation between miR-4728-3p and its targets using RNAduplex [23]. Figure 1F shows the distribution of duplex energies of 3′ UTRs with IS 6-mer matches comparing down regulated genes with those carrying IS matches but unaffected by miR-4728-3p overexpression. Setting an arbitrary threshold, we examined the IS-target hybrids with highest stability and found only one example of a 10 nt uninterrupted base pairing that included the IS match (Fig. S2 in File S1). This shows that the IS motif is not part of a longer regulatory motif region and that an IS match alone, again in analogy to a typical CS match, is sufficient to direct repression of a target.

### Physical interaction between IS and target RNA

**Figure 2:**
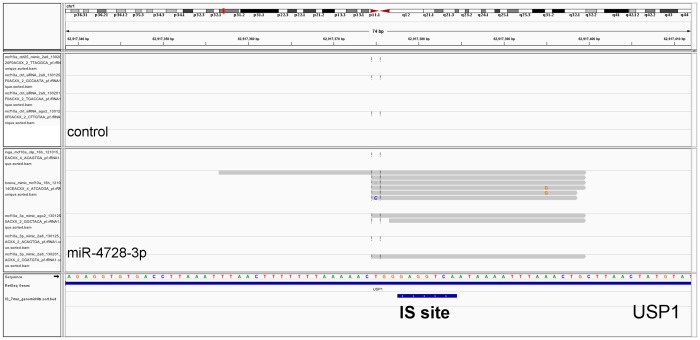
IGV view (Integrative Genomics Viewer) showing the enrichment of AGO-CLIP sequencing reads in miR-4728-3p transfected vs non-transfected control around the IS target site on the 3′UTR of the Ubiquitin carboxyl-terminal hydrolase 1 (USP1) gene. The positions of the 7- and 6-mer IS target sites are highlighted below the USP1 gene.

To confirm the physical interaction between IS and target mRNA, we applied AGO-CLIP to MCF 10A cells transfected with miR-4728-3p mimic and matched controls. Since the down regulation profiles were obtained 32 hours post-transfection, we reasoned that the physical association had to be a preceding event and harvested the cells at 16 hours. Immunoprecipitations were prepared with AGO2-specific antibodies or the pan-AGO antibody 2A8. Sequence reads were aligned to the human genome and interaction peaks were defined as all reads mapping to seed matches and extending 10 nt in either direction. Peaks were selected as positive for miR-4728-3p, if the reads were enriched in the transfection experiments compared to the matched controls. A total of 777 regions were associated with either CS (nt 2–7, n = 449) or IS (nt 6–11, n = 328) miR-4728-3p binding. Figure 2 shows ubiquitin carboxyl-terminal hydrolase 1 (USP1) as an example of IS binding in the 3′UTR. Twenty-four 3′ UTRs were found to contain both types of seed target sites, which tended to be proximal or even overlapping.

The cells for AGO-CLIP were harvested 16 hours earlier than for the microarray studies. Regardless, we found that 26% of transcripts with 3′ UTRs containing IS match peaks in the AGO-CLIP data were also down regulated in the array experiment, compared to 30% for CS targets. In accordance with other studies, the majority of peaks fall into coding sequences (CDS). However, as observed previously by Rudensky and co-workers [16], interaction with the CDS does not seem to lead to down regulation of targets and accordingly, enrichment analysis with SylArray performed on CDS found no overrepresentation of either seed (Fig. S3 in File S1).

### IS usage beyond miR-4728-3p

**Figure 3:**
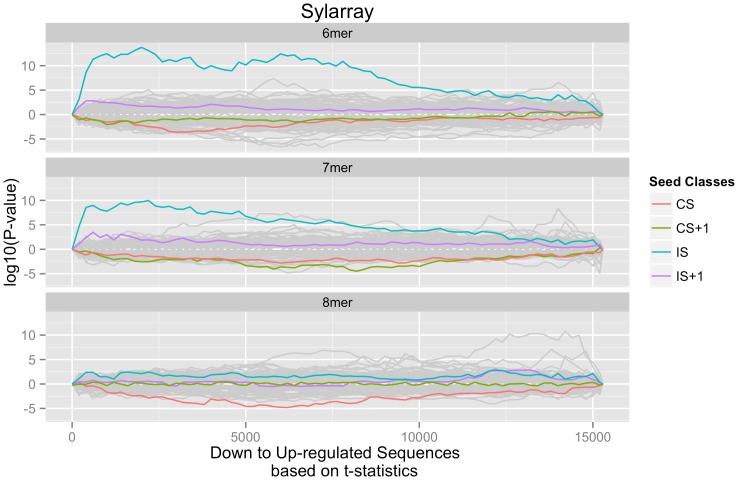
IS targeting is not restricted to miR-4728-3p. SylArray enrichment landscape from a microarray experiment of miR-30a overexpression in HCT116 cells. Data generated by Baraniskin *et al.* was analyzed by SylArray. Word designations and graph details as in Fig. 1a, with CS and IS referring to corresponding miR-30a positions.

Previous studies have only identified over-representation of CS type sites among down regulated genes detected by microarray profiling following miRNA transfection [25]. Thus, we wanted to test the validity of our observations by investigating independent data sets. During the preparation of this manuscript, Tollervey and co-workers [27] reported an elegant characterization of AGO1-bound miRNA interactions using crosslinking, ligation, and sequencing of hybrids (CLASH), where the target site is identified together with its cognate miRNA by the formation of RNA chimeras, allowing unambiguous positioning of the region of interaction in the miRNA. We searched this data for interactions comprising the IS region and found that 43 out of 400 detected miRNAs (11%) were shown to be able to interact with their IS region. Overall, however, this CLASH data suggests that IS interactions are not common since they represented less than 1% of all interactions identified in the study. The list of miRNAs that utilize the IS region comprises clinically important miRNAs that have been shown to act as oncogenes or tumor suppressors such as miR-15b, miR-16, miR-17, miR-92a and miR-106b. To test whether the IS interactions detected for these miRNAs were functional, we downloaded existing expression data sets of miRNA manipulation studies from the GEO database and searched for enriched motifs with SylArray. While some data sets showed only moderate enrichment for the IS sites, a clear example of this interaction was miR-30a. Here, overexpression experiments relied on endogenous processing of miR-30a, a miRNA that has been extensively studied and whose 5′ end processing is so well characterized that its precursor has been used as backbone in many miRNA expression vectors. When examining the reported miR-30a overexpression data [28] (GEO accession #GSE29921) we found that the strongest signal corresponded to the IS match at positions 6–12 (Fig. 3), demonstrating that the functionality of the IS region is not exclusive for miR-4728-3p.

### miR-4728-3p IS regulation connects the HER2 and ESR1 pathways

**Figure 4:**
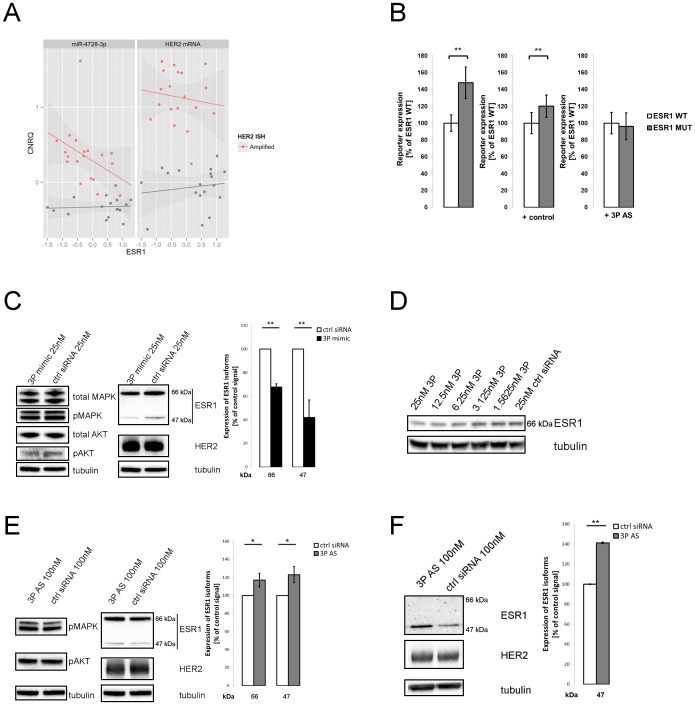
miR-4728-3p IS regulates ESR1. **A.** qRT-PCR analysis of ESR1 and HER2 transcripts and miR-4728-3p among a panel of 38 breast cancer tumors (19 HER2+, 19 HER2-). Calibrated Normalized Relative Quantity (CNRQ) of miR-4728-3p (left) and HER2 (right) is plotted against expression levels of ESR1. Tumors classified as HER2+ by ISH are shown in red, HER2- in grey. Expression was normalized to a panel of reference genes. For details see text and material and methods. **B.** Luciferase assay in BT-474 with ESR1 3′UTR constructs carrying either wild type target site of miR-4728-3p internal seed (WT) or mutated internal seed site (MUT). Firefly luciferase activity was normalized against Renilla luciferase. Reporter activity is given as % of WT in respective experiment. Repression of WT ESR1 construct by endogenous miR-4728-3p (left) is alleviated by an antisense oligo (AS) against endogenous miRNA (right) but not by a non-targeting control (middle). **C.** Western blot (left) and protein quantification (right) of ESR1 in MCF7. The two main isoforms of ESR1 (47 and 66 kDa), plotted as percentage of control signal of matching size, are down regulated upon transfection of miR-4728-3p mimics. Levels of HER2, (p)MAPK and (p)AKT remain largely unchanged. **D.** MCF7 cells were transfected with indicated concentrations of miR-4728-3p mimic. ESR1 levels show a concentration-dependent down-regulation that is most pronounced at highest tested concentration (25 nM). **E.** Western blot (left) and protein quantification (right) of ESR1 in BT474. ESR1 is up regulated when blocking endogenous miR-4728-3p with AS-oligonucleotides, while pMAPK and pAKT remain largely unchanged. **F.** Western blot (left) and protein quantification (right) of ESR1 in HCC1954 cells. ESR1 isoform of 47 kDa is up regulated under miR-4728-3p blocking. The main 66 kDa isoform is not detectable in this ER- cell line. Signals were quantified with ImageJ and normalized to total protein by Coomassie stain. Tubulin was used as a loading control. Asterisks denote p-values of <0.05 (*), and <0.005 (**) in Student′s t-test.

miR-4728-3p is encoded within one of the most important BC oncogenes, so we wanted to evaluate the clinical relevance of target gene regulation by the IS. Since CS and IS seem to differ only by their shifted position in relation to the 5′ end of the miRNA, we tested if target prediction algorithms designed for CS could also detect IS matches. We used TargetScan Custom 5.2 [12] and submitted the 7-mer miR-4728-3p IS as seed sequence. Among 104 predicted targets (Table S1) we found USP1, the IS interacting mRNA detected by the AGO-CLIP experiment described above (Fig. 2). Interestingly, we also observed a predicted IS match in the 3′ UTR of ESR1 close to the polyadenylation site (NM_000125), one of the preferred locations for canonical target sites [29]. This is of special interest since HER2 and ESR1 expression have been observed to be anti-correlated in HER2+ tumors [30]. We therefore measured the expression of miR-4728-3p, HER2 and ESR1 in a set of 19 Her2- and 19 Her2+ breast tumors by qPCR. Anti-correlation of HER2 and ESR1 could not be confirmed in our small sample set (Spearman rho −0.149, p = 0.541) but, interestingly, anti-correlation was evident between miR-4728-3p and ESR1 (Spearman rho −0.495, p = 0.033) (Fig. 4A), hinting at the possibility of transcription independent of its host gene or a specific regulated processing. Conventional bioinformatic predictions using TargetScan and DIANA micro-T [31] failed to detect any miR-4728-3p CS target sites in ESR1 and deep sequencing of HER2+ BC tumors showed no evidence of 5′-end truncated isomiRs (Fig. 1C), so we searched our AGO-CLIP data for support for an IS-ESR1 interaction. MCF 10A expresses very low endogenous levels of ESR1, complicating the analysis (Fig. S4 in File S1). Moreover, the IS match is proximal to a predicted mir-26a/b CS target site. Although these miRNAs are expressed at very low levels in MCF 10A cells, the association of sequencing reads to either of the two miRNA target sites is ambiguous. Still, all reads aligning to the ESR1 3' UTR were indeed found in the region of the predicted IS match (Fig. S5 in File S1).

To investigate the regulation of ESR1 by the miR-4728-3p IS target site experimentally, we cloned a fragment of the ESR1 3' UTR containing the target region downstream of a Firefly luciferase reporter gene. Luciferase activity was normalized to Renilla luciferase and compared to an analogous vector where the IS match of miR-4728-3p was mutated from GAGGTCA to CTCCAGT. The vectors were transfected into the miR-4728-3p expressing, HER2+/ER+ cell line BT-474, to test if endogenously expressed miRNAs could use the IS and regulate the reporter. We checked the correct 5′-end processing of miR-4728-3p in these cells by deep sequencing of RNAs co-immunoprecipitated with AGO2 (Fig. 1C). Figure 4B shows that substitution of the IS site in the reporter plasmid resulted in increased luciferase activity, indicating that this single sequence mediates repression of the reporter gene. To prove that this repression is associated with miR-4728-3p, we blocked the endogenous miR-4728-3p with 2′-O-methyl antisense oligonucleotides which released the repression of the ESR1 3' UTR as expected (Fig. 4B, right).

Repression of ESR1 3′UTR by miR-4728-3p indicates a new mechanism for cross-talk between the HER2 and estrogen pathways. We therefore proceeded to validate the results of the reporter assays at ESR1 protein level. Western blotting showed that overexpression of miR-4728-3p in Her2-/ER+ MCF7 cells decreased ESR1 protein compared to transfection with a negative control (Fig. 4C). The effect was observed in both the 66 kDa and the 47 kDa isoforms that share the same 3′UTR (∼30% and ∼ 60% decrease respectively) and was concentration-dependent (Fig. 4D). In accordance with these results, blocking endogenous miR-4728-3p with antisense 2′-O-methyl oligonucleotides in HER2+/ER+ BT-474 and HER2+/ER- HCC1954 cells (both endogenously expressing miR-4728-3p, see Fig. S4 in File S1) resulted in an increase of ESR1 protein levels (Fig. 4E and 4F). In HCC1954 cells only the 47 kDa ESR1 isoform was detected. Here, miR-4728-3p down-regulates ESR1 to around 70% (Fig. 4F). ESR1 protein levels have been reported to be regulated by HER2 overexpression through downstream targets such as MAPK1 and AKT1 [32]. To exclude that the observed down regulation of ESR1 proceeds through these pathways rather than by miR-4728-3p-mediated repression, we assessed total HER2 expression as well as levels of activated pMAPK1 and pAKT in our experiments. Total amounts of HER2, pMAPK1 and pAKT1 mostly remain unchanged at this time point (Fig. 4D and 4E, left) and any observed changes rather indicated a slight decrease in MAPK1 upon miR-4728-3p up regulation. These results show that the regulation of ESR1 does not proceed through an indirect effect on these pathways and confirm that a miR-4728-3p IS interaction functionally connects the two major BC biomarkers.

## Discussion

We have demonstrated that the *HER2* gene is a bi-functional locus. It encodes the growth factor receptor and regulates target genes through its embedded miRNA. This work provides new insight into the role of HER2 in carcinogenesis. In turn, it led us to the discovery of a new mechanism for miRNA action. We have shown that miR-4728-3p can use positions 6-12 instead of the canonical 2-8 seed sequence for interaction with its targets. The position of the IS with respect to the 5' end suggests structural constraints. Aligning the IS regions of miR-4728-3p and other miRNAs from published data failed to identify a clear IS consensus sequence. This indicates that, just as for CS sites, any nucleotide sequence could function as an IS and no specific molecular interaction between specific nucleotides and AGO is required. Structural studies propose that AGO proteins prearrange the seed region in an A-form conformation, exposing the edges of the CS bases of the guide RNA to the solvent to better anneal to the target [33]. The structure of the CS in the complex is disrupted in the IS region where solvent exposure of the bases is not maximized for interaction [34]. The most common miR-4728-3p isomiRs in HER2+ cells are 24-26 nt in length with the longest isomiR ending in a non-templated U, probably the product of post-transcriptional uridylation [35]. Therefore, it may be possible that after anchoring to the MID and PAZ domains, such a long guide RNA may be forced to shift position in the RNA groove.

Exceptions to the seed rule have been observed before, but the biological consequences of these interactions have only been described in detail in very few cases including, for instance, extensive centered base pairing [26] or randomly distributed pairing over the whole miRNA length as in the case of miR-24 [18]. Unlike all these examples, the fixed position with respect to the 5' end of the miRNA could make the IS amenable for bioinformatic predictions, as the ESR1 and USP1 examples described here show. In case these predicted interactions are verified, even if it can be anticipated that they are rare, it may be possible uncover a number of unexpected connections between cancer-related miRNAs and different well-known oncogenes or tumor suppressors. As an example, mir-15a IS is predicted to target PTEN; mir-30a could interact with ERBB4 or FOXO1; mir-106b with CAV1 or FOXO3, etc.

Regardless of these speculations, the proven IS activity of miR-4728-3p may have interesting clinical implications. We show here that endogenous miR-4728-3p regulates ESR1 and that the long isoforms responsible for this IS interaction are the most common isomiRs found in HER2+ BC cell lines. We speculated whether this observation could be important for actual BC tumors. When analyzing small RNA sequencing data generated in our laboratory in the context of a parallel project, we found that the long 24-26 nt isomiRs are also consistently the most common isoforms expressed in HER2+ tumors (Fig. 1C). As mentioned above, it has previously been observed that HER2 and ESR1 expression tend to be inversely correlated. Consequently, the fraction of ER+ tumors is lower among HER2+ tumors compared to HER2-. Additionally, HER2+/ER+ tumors consistently display lower ESR1 expression levels than ER+ tumors outside this group. Protein and RNA quantitation analyses of primary tumor samples have shown that HER2 and ESR1 expression are anti-correlated in HER2+ tumors, while they tend to be uncorrelated or even positively correlated in HER2- tumors [36]. Here we show that *HER2* amplification may lead to ESR1 down-regulation through the IS activity of miR-4728-3p and that miR-4728-3p is even more anti-correlated to ESR1 than is HER2 in our sample set. Furthermore, this anti-correlation is more pronounced among HER2+ tumors, which is probably the consequence of miR-4728-3p reaching the concentration levels required for target suppression. This is particularly important, since HER2 amplification is believed to be a mechanism of developing endocrine therapy resistance. Several clinical and preclinical studies have found HER2 amplification to be associated with poor outcome in patients treated with tamoxifen, indicating that a mechanistic interconnection of the two pathways may have clinical consequences [36], [37]. Hyper-activation of the HER2 receptor leads to negative regulation of FOXO3a, a transcription factor controlling ESR1 through PI3K/AKT signaling [38] and HER2-induced MAPK activation as well as carboxy-terminal HER2 fragments have been shown to be implicated in ESR1 down regulation [39]. Although sufficient to affect ESR1, miR-4728-3p activity exerts a limited effect on expression levels of estrogen responsive genes in our experimental set up (data not shown). This suggests that while miR-4728-3p activity alone may not result in tamoxifen resistance, it adds an additional layer of regulation to the highly complex interplay of ESR1 and HER2 in BC.

## Supporting Information

Table S1 **List of predicted IS targets.**
(PDF)

Table S2 **List of primer sequences used to generate qPCR/qRT-PCR expression data.**
(PDF)

File S1 **A text file containing Supporting Figures S1, S2, S3, S4 and S5.**
(DOCX)
