# Supplementary material for: The HER2-Encoded miR-4728-3p Regulates ESR1 through a Non-Canonical Internal Seed Interaction
Source: PLoS One. 2014 May 14;9(5):e97200. doi: 10.1371/journal.pone.0097200 (PMC4020767; doi:10.1371/journal.pone.0097200)
Supplement: Table S1 — List of predicted IS targets. (PDF) [file pone.0097200.s001.pdf]

Target gene	Gene name	Conserved sites			
		total	8mer	7mer-m8	7mer-1A
SMG1	PI-3-kinase-related kinase SMG-1	2	1	0	1
SSR3	signal sequence receptor, gamma (translocon-associated protein gamma)	2	1	0	1
TNNI1	troponin I type 1 (skeletal, slow)	2	1	0	1
APLN	apelin	1	1	0	0
BCL11B	B-cell CLL/lymphoma 11B (zinc finger protein)	1	1	0	0
C1orf43	chromosome 1 open reading frame 43	1	1	0	0
CNTNAP1	contactin associated protein 1	1	1	0	0
CRTC2	CREB regulated transcription coactivator 2	1	1	0	0
CSF1R	colony stimulating factor 1 receptor	1	1	0	0
DDX54	DEAD (Asp-Glu-Ala-Asp) box polypeptide 54	1	1	0	0
FLJ11151	hypothetical protein FLJ11151	1	1	0	0
GBA2	glucosidase, beta (bile acid) 2	1	1	0	0
GOSR1	golgi SNAP receptor complex member 1	1	1	0	0
IGF2	insulin-like growth factor 2 (somatomedin A)	1	1	0	0
IKZF2	IKAROS family zinc finger 2 (Helios)	1	1	0	0
LEP	leptin	1	1	0	0
LOXL3	lysyl oxidase-like 3	1	1	0	0
MEOX1	mesenchyme homeobox 1	1	1	0	0
MEX3A	mex-3 homolog A (C. elegans)	1	1	0	0
MPP2	membrane protein, palmitoylated 2 (MAGUK p55 subfamily member 2)	1	1	0	0
MRFAP1L1	Morf4 family associated protein 1-like 1	1	1	0	0
MRPL11	mitochondrial ribosomal protein L11	1	1	0	0
MYADM	myeloid-associated differentiation marker	1	1	0	0
OSM	oncostatin M	1	1	0	0
PDLIM2	PDZ and LIM domain 2 (mystique)	1	1	0	0
PIB5PA	phosphatidylinositol (4,5) bisphosphate 5-phosphatase, A	1	1	0	0
PPP2R5D	protein phosphatase 2, regulatory subunit B', delta isoform	1	1	0	0
PURB	purine-rich element binding protein B	1	1	0	0
RAB11FIP1	RAB11 family interacting protein 1 (class I)	1	1	0	0
RAG1	recombination activating gene 1	1	1	0	0
RP1L1	retinitis pigmentosa 1-like 1	1	1	0	0

SCRT2	scratch homolog 2, zinc finger protein (Drosophila)	1	1	0	0
SEMA3E	sema domain, immunoglobulin domain (Ig), (semaphorin) 3E	1	1	0	0
SETX	senataxin	1	1	0	0
SLC30A3	solute carrier family 30 (zinc transporter), member 3	1	1	0	0
SLC30A8	solute carrier family 30 (zinc transporter), member 8	1	1	0	0
SLC39A10	solute carrier family 39 (zinc transporter), member 10	1	1	0	0
SMURF1	SMAD specific E3 ubiquitin protein ligase 1	1	1	0	0
SUSD1	sushi domain containing 1	1	1	0	0
TLR4	toll-like receptor 4	1	1	0	0
TNPO1	transportin 1	1	1	0	0
USP1	ubiquitin specific peptidase 1	1	1	0	0
ZNF24	zinc finger protein 24	1	1	0	0
ZNF592	zinc finger protein 592	1	1	0	0
ZNF629	zinc finger protein 629	1	1	0	0
ATRN	attractin	1	0	1	0
BTG1	B-cell translocation gene 1, anti-proliferative	1	0	1	0
CALN1	calneuron 1	1	0	1	0
CD28	CD28 molecule	1	0	1	0
DYRK1A	dual-specificity tyrosine-(Y)-phosphorylation regulated kinase 1A	1	0	1	0
FZD4	frizzled homolog 4 (Drosophila)	1	0	1	0
GABRE	gamma-aminobutyric acid (GABA) A receptor, epsilon	1	0	1	0
IMPDH1	IMP (inosine monophosphate) dehydrogenase 1	1	0	1	0
KCNE3	potassium voltage-gated channel, Isk-related family, member 3	1	0	1	0
KRT76	keratin 76	1	0	1	0
MARK4	MAP/microtubule affinity-regulating kinase 4	1	0	1	0
NAV1	neuron navigator 1	1	0	1	0
PBX1	pre-B-cell leukemia homeobox 1	1	0	1	0
SDC1	syndecan 1	1	0	1	0
SLC45A3	solute carrier family 45, member 3	1	0	1	0
SUV39H1	suppressor of variegation 3-9 homolog 1 (Drosophila)	1	0	1	0
TERF2	telomeric repeat binding factor 2	1	0	1	0
TMEM132B	transmembrane protein 132B	1	0	1	0
**ESR1**	**estrogen receptor 1**	1	0	0	1

ARFIP2	ADP-ribosylation factor interacting protein 2 (arfaptin 2)	1	0	0	1
C10orf104	chromosome 10 open reading frame 104	1	0	0	1
C15orf55	chromosome 15 open reading frame 55	1	0	0	1
C3orf10	chromosome 3 open reading frame 10	1	0	0	1
CALCR	calcitonin receptor	1	0	0	1
CNNM3	cyclin M3	1	0	0	1
DDX3X	DEAD (Asp-Glu-Ala-Asp) box polypeptide 3, X-linked	1	0	0	1
DENND1A	DENN/MADD domain containing 1A	1	0	0	1
EIF5A2	eukaryotic translation initiation factor 5A2	1	0	0	1
ELMO2	engulfment and cell motility 2	1	0	0	1
FOXA1	forkhead box A1	1	0	0	1
GATAD2B	GATA zinc finger domain containing 2B	1	0	0	1
ITCH	itchy E3 ubiquitin protein ligase homolog (mouse)	1	0	0	1
LIMD1	LIM domains containing 1	1	0	0	1
LYRM1	LYR motif containing 1	1	0	0	1
MRFAP1	Mof4 family associated protein 1	1	0	0	1
NPTXR	neuronal pentraxin receptor	1	0	0	1
ORMDL3	ORM1-like 3 (S. cerevisiae)	1	0	0	1
PIP4K2B	phosphatidylinositol-5-phosphate 4-kinase, type II, beta	1	0	0	1
PLAGL2	pleiomorphic adenoma gene-like 2	1	0	0	1
PLEKHH3	pleckstrin homology domain containing, family H member 3	1	0	0	1
PML	promyelocytic leukemia	1	0	0	1
RAB6A	RAB6A, member RAS oncogene family	1	0	0	1
RC3H1	ring finger and CCCH-type zinc finger domains 1	1	0	0	1
RECQL5	RecQ protein-like 5	1	0	0	1
SH3PXD2A	SH3 and PX domains 2A	1	0	0	1
SH3TC2	SH3 domain and tetratricopeptide repeats 2	1	0	0	1
SHANK2	SH3 and multiple ankyrin repeat domains 2	1	0	0	1
SLC10A7	solute carrier family 10, member 7	1	0	0	1
SLC5A12	solute carrier family 5 (sodium/glucose cotransporter), member 12	1	0	0	1
SMYD3	SET and MYND domain containing 3	1	0	0	1
SNX27	sorting nexin family member 27	1	0	0	1
SRGAP3	SLIT-ROBO Rho GTPase activating protein 3	1	0	0	1

STK4	serine/threonine kinase 4	1	0	0	1
SYNGR1	synaptogyrin 1	1	0	0	1
SYS1	SYS1 Golgi-localized integral membrane protein homolog (S. cerevisiae)	1	0	0	1
TNFAIP1	tumor necrosis factor, alpha-induced protein 1 (endothelial)	1	0	0	1
XPO4	exportin 4	1	0	0	1
ZDHHC16	zinc finger, DHHC-type containing 16	1	0	0	1
ZNF648	zinc finger protein 648	1	0	0	1
