# Supplementary material for: The HER2-Encoded miR-4728-3p Regulates ESR1 through a Non-Canonical Internal Seed Interaction
Source: PLoS One. 2014 May 14;9(5):e97200. doi: 10.1371/journal.pone.0097200 (PMC4020767; doi:10.1371/journal.pone.0097200)
Supplement: Table S2 — List of primer sequences used to generate qPCR/qRT-PCR expression data. (PDF) [file pone.0097200.s002.pdf]

primer name	target gene	
ACTB.F	Actin	GATGACCCAGATCATGTTTGAG
ACTB.R	Actin	CTGGATAGCAACGTACATGG
RN7SL.R	Homo sapiens RNA, 7SL, cytoplasmic	CAGGTCCAGTTTTTTTTTTTTTTTAGAGACG
RN7SL.F	Homo sapiens RNA, 7SL, cytoplasmic	GAATAGCCACTGCACTCCAG
RNU6.R	Homo sapiens RNA, U6 small nuclear	CAGGTCCAGTTTTTTTTTTTTTTTAAAAATATGGAA
RNU6.F	Homo sapiens RNA, U6 small nuclear	CGCAAGGATGACACGCAAATTC
SNORD48.F	Homo sapiens small nucleolar RNA, C/D box 48	GTGATGATGACCCCAGGTAACTC
SNORD48.R	Homo sapiens small nucleolar RNA, C/D box 48	CAGGTCCAGTTTTTTTTTTTTTTTGGTCAGA
let7a.F	hsa-let-7a	GCAGTGAGGTAGTAGGTTGT
let7a.R	hsa-let-7a	GGTCCAGTTTTTTTTTTTTTTTAACTATAC
miR.191.5p.F	hsa-miR-191 5p	CGCAGCAACGGAATCCCAAA
miR.191.5p.R	hsa-miR-191 5p	AGGTCCAGTTTTTTTTTTTTTTTCAGCTGCT
ESR1.ex.1.2.F	Estrogen receptor alpha exon junction 1-2	GCATTCTACAGGCCAAATTCA
ESR1.ex.1.2.R	Estrogen receptor alpha exon junction 1-2	ACTGGCCAATCTTTCTCTGC
ESR1.ex.7.8.F	Estrogen receptor alpha exon junction 7-8	CCTCATCCTCTCCCACATCA
ESR1.ex.7.8.R	Estrogen receptor alpha exon junction 7-8	AGATGCTCCATGCCTTTGTT
ESR1.3UTR.F	Estrogen receptor alpha 3' UTR	ACACAGACCCCTTTGCATTC
ESR1.3UTR.R	Estrogen receptor alpha 3' UTR	TGTAGTGCACAAAAAGCATTG
ERBB2.exon2.3.F	Receptor tyrosine-protein kinase erbB-2 exon junction 2-3	GTCCTTCCTGCAGGATATCCA
ERBB2.exon2.3.R	Receptor tyrosine-protein kinase erbB-2 exon junction 2-3	CACTTGGTTGTGAGCGATGAG
ERBB2.ex.23.24.F	Receptor tyrosine-protein kinase erbB-2 exon junction 23-24	CCCATCTGCACCATTGATGTC
ERBB2.ex.23.24.R	Receptor tyrosine-protein kinase erbB-2 exon junction 23-24	GAGTCAATCATCCAACATTTGACC
