# Supplementary material for: The HER2-Encoded miR-4728-3p Regulates ESR1 through a Non-Canonical Internal Seed Interaction
Source: PLoS One. 2014 May 14;9(5):e97200. doi: 10.1371/journal.pone.0097200 (PMC4020767; doi:10.1371/journal.pone.0097200)
Supplement: File S1 — A text file containing Supporting Figures S1, S2, S3, S4 and S5. (DOCX) [file pone.0097200.s003.docx]

**SUPPORTING INFORMATION**

**The HER2-encoded miR-4728-3p regulates ESR1 through a non-canonical internal seed**

Inga Newie, Rolf Soekilde, Helena Persson, Dorthe Grabau, Natalia Rego, Anders Kvist, Kristoffer von Stedingk, Håkan Axelson, Åke Borg, Johan Vallon-Christersson and Carlos Rovira

**Supporting Figure Legends**

**Supporting Figure S1**

miR-1 mimic transfection shows expected regulation of its targets with the canonical seed. SylArray enrichment landscape plots for 6-, 7- and 8-mer words (from top to bottom) for ranked genes from a microarray experiment of miR-1 overexpression in MCF 10A. 3’ UTRs are sorted on the x-axis from most down regulated (left) to most up regulated (right). Significance of the words is given as log10(P-value) with positive values corresponding to enrichment and negative values to depletion. Highlighted are words corresponding to canonical seed (CS, red), CS shifted by one base towards the miRNA’s 3’ end (CS+1, green), internal seed (IS, blue) and IS +1 (purple). Most highly enriched word is the 7-mer CS.

**Supporting Figure S2**

Predicted base paring between mir-4728-3p internal seed and targets displaying the

most stable RNAduplex scores as calculated with the Vienna RNA package RNA- duplex function.

**Supporting Figure S3**

Interaction with the CDS does not lead to down regulation of targets. SylArray enrichment landscape plots for 6-, 7- and 8-mer words (from top to bottom) for ranked genes from a microarray experiment of miR-4728-3p overexpression in MCF 10A. 3’ UTRs are sorted on the x-axis from most down regulated (left) to most up regulated (right). Significance of the words is given as log10(P-value) with positive values corresponding to enrichment and negative values to depletion. Highlighted are words corresponding to canonical seed (CS+1, green) and internal seed (purple) shifted by one base towards the miRNA’s 3’ end. Not all seeds are included in the figure, due to the thresholds of the SylArray algorithm on p-values. The CS and IS seeds are non-significant and therefore not found in the output from SylArray. No target enrichment can be observed among the coding sequences (CDS).

**Supporting Figure S4**

Expression levels of selected genes in investigated cell lines. BT474 (left), HCC1954 (middle) and MCF 10A (right) were cultured under standard conditions (see material and methods). TRIZOL was used to extract RNA, which was DNase-treated before reverse transcription. Amplification with primers given in supporting table 2 was monitored with SYBR green reagent. Expression levels of ESR1 (light grey) and HER2 (dark grey) were normalized to GAPDH, while miR-4728-3p expression (black) was adjusted to let-7a and GAPDH levels (assuming an assay efficiency of 100% for all reactions). HER2 and ESR1 status of investigated cell lines correspond to expression levels reported in literature.

**Supporting Figure S5**

Alignment of AGO-CLIP reads from mir-4728-3p transfected MCF10A cells to the ESR1 3’UTR. Alignment of sequencing reads within ESR1 from AGO-CLIP of miR-4728-3p transfected MCF 10A shown in UCSC genome browser. Upper panel contains full length ESR1. Reads are only detected in a very narrow area magnified in the lower panel. Binding sites for miR-26a/b and IS of miR-4728-3p are boxed.

**Supporting Figure S1**

**Supporting Figure S2**

ILMN_2273224

target 5' AAUCA A UCA A 3'

GG GCAG GG GGAGGUCA

CC CGUC CU CCUCCAGU

miRNA 3' GA C C CGUAC 5'

ILMN_2273224

target 5' CAG UCA AGCC 3'

AGG GGAGGUCA

UCC CCUCCAGU

miRNA 3' GACCCCG UC CGUAC 5'

ILMN_1741841

target 5' A A C U C 3'

G GGU G GGGGGA

C CCG C CUCCCU

miRNA 3' GA C U CCAGUCGUAC 5'

ILMN_1741841

target 5' U UA U U 3'

GGCAG GG GAGGUCG CAUG

CCGUC CU CUCCAGU GUAC

miRNA 3' GACC CC C 5'

ILMN_1735827

target 5' CU CAA U U 3'

U AG GGAGGUC

G UC CCUCCAG

miRNA 3' GACCCC UCC UCGUAC 5'

ILMN_1696975

target 5' UAAA ACU AAUAAAAUUU 3'

A GGGAGGUCA

U CCCUCCAGU

miRNA 3' GACCCCG CCU CGUAC 5'

ILMN_1707513

target 5' C CCU C C 3'

CAG GGGAGGUC AG AUG

GUC CCCUCCAG UC UAC

miRNA 3' GACCCC CU G 5'

ILMN_1707513

target 5' CACCCA U G 3'

GGGU GAGGUC GGU

CCCG CUCCAG UCG

miRNA 3' GAC UCCUCC UAC 5'

ILMN_1671554

target 5' AC U UCU UC A AA 3'

C GGA GAGGUC UG G

G CCU CUCCAG GU C

miRNA 3' GACCCC U CC UC A 5'

ILMN_1671554

target 5' U UGCU UGU G 3'

CAG GA GAGGUCA GC UG

GUC CU CUCCAGU CG AC

miRNA 3' GACCCC CC U 5'

**Supporting Figure S3**

**Supporting figure S4**

**Supporting Figure S5**
